# Medullary Thyroid Carcinoma in Patients with Graves’ Disease—A Case Series and Literature Review

**DOI:** 10.3390/jcm13154391

**Published:** 2024-07-27

**Authors:** Oana Popa, Melania Balaș, Ioana Golu, Daniela Amzăr, Flore Varcuș, Mărioara Cornianu, Mihaela Iacob, Valentin-Tudor Popa, Mihaela Vlad

**Affiliations:** 1 Center of Molecular Research in Nefrology and Vascular Disease, Department of Endocrinology, University of Medicine and Pharmacy “Victor Babeș” Timișoara, 300041 Timișoara, Romania; oana.taban@umft.ro (O.P.); golu.ioana@umft.ro (I.G.); amzar.daniela@umft.ro (D.A.); vlad.mihaela@umft.ro (M.V.); 2 Department of Surgery II, University of Medicine and Pharmacy “Victor Babeș” Timișoara, 300041 Timișoara, Romania; varcus.florian@yahoo.com; 3 Anapatmol Research Center, Department of Pathology, University of Medicine and Pharmacy “Victor Babeș” Timișoara, 300041 Timișoara, Romania; cornianu.marioara@umft.ro (M.C.); morfopat@umft.ro (M.I.); 4 Morphoderm Research Center, Department of Dermatology, University of Medicine and Pharmacy “Victor Babeș” Timișoara, 300041 Timișoara, Romania

**Keywords:** medullary thyroid carcinoma, Graves’ disease, calcitonin, screening

## Abstract

Introduction: Graves’ disease (GD) is an autoimmune disorder affecting the thyroid gland, leading to systemic manifestations such as hyperthyroidism, Graves’ orbitopathy, and pretibial myxedema. Contrary to previous beliefs that hyperthyroidism protects against thyroid cancer, recent studies reveal an increased incidence of thyroid malignancies in GD patients, particularly differentiated thyroid carcinomas and, in rare cases, medullary thyroid carcinoma (MTC). Case series: This case series presents three female GD patients diagnosed with MTC, highlighting the complexities of diagnosis and management. All patients exhibited thyroid nodules with suspicious ultrasonographic features, elevated plasma calcitonin levels, and required total thyroidectomy. Histological examination confirmed MTC. Discussion: These cases underscore the importance of routine calcitonin screening in GD patients with thyroid nodules to facilitate early detection and improve prognosis. Our findings suggest that while the coexistence of GD and MTC is likely incidental, vigilant monitoring and comprehensive evaluation are crucial for timely intervention. Conclusions: This study advocates for integrating calcitonin testing into the standard diagnostic protocol for GD patients presenting with thyroid abnormalities.

## 1. Introduction

Graves’ disease (GD) is an autoimmune condition affecting the thyroid gland, with systemic manifestations involving the heart, skeletal muscles, skin, and bones. The pathogenesis of the disease involves the production of thyroid-stimulating antibodies (TRAbs) that activate the thyroid-stimulating hormone receptor on thyroid cells, triggering thyroid hormone synthesis. Other manifestations of the disease, such as Graves’ orbitopathy, pretibial myxedema, and thyroid acropathy, are caused by inflammation, cellular proliferation, and the increased growth of connective and adipose tissues due to the actions of TRAbs and cytokines released by cytotoxic T lymphocytes that stimulate fibroblast growth [1].

The reported prevalence of hyperthyroidism is 0.8% in the USA and 1.3% in Europe [2]. In iodine-sufficient areas, GD accounts for the majority of cases in most epidemiological studies [3], with as many as 20–30 annual cases per 100.000 individuals [4,5]. The condition is more frequent in female patients than in males. Epidemiological studies have reported that 3% of women and 0.5% of men develop this condition during their lifetime [6], mostly during adulthood between the ages of 30 and 60 [7].

Contrary to the former belief that hyperthyroidism acts as a protective mechanism against thyroid cancer [8], recent studies have shown a higher incidence and risk of thyroid cancer in GD patients. The prevalence of thyroid cancer in these patients may reach up to 17% of cases, mostly consisting of differentiated thyroid carcinomas [6].

Medullary thyroid carcinoma (MTC) is a notably aggressive form of thyroid cancer. It occurs sporadically in 75% of cases. The remaining 25% of cases are inherited, often presenting as part of Multiple Endocrine Neoplasia Syndrome (MEN) types 2A and 2B, as well as in familial MTC patients. MTC accounted for 0.6% of all thyroid carcinomas in GD patients. A systematic review of the literature revealed an extremely rare coexistence between GD and MTC, with only 15 cases reported. This occurrence seems to be incidental, without any definite factors involved such as the patient’s age, gender, or tumor size [6,9]. Increased plasma calcitonin levels are highly suggestive in patients with suspicious thyroid nodules on ultrasound [10]. Because MTC is often asymptomatic in the initial stages of the disease and GD may mask the diagnosis, early detection of MTC in GD patients is often extremely challenging [6].

In this article, we aim to present a case series of three patients with GD who were diagnosed and treated for MTC and to discuss further recommendations that could help for an early diagnosis of MTC in GD patients.

## 2. Materials and Methods

All cases were investigated and diagnosed in the Department of Endocrinology of the County Hospital Timișoara, Romania. Anterior neck ultrasounds were performed using Esaote Mylab Seven ultrasound machine (Esaote Mylab Seven, Genoa, Italy) with a 15 MHz linear probe. Thyroid nodules were graded according to the ACR TIRADS classification. Laboratory assays were performed in the Laboratory Department of our hospital. The methodology for immunological analysis was enhanced chemiluminescence immunoassay method for TSH, FT3, and FT4, chemiluminescence microparticle immunoassay for antithyroid peroxidase antibodies (ATPO) and antithyroglobulin antibodies (ATG), chemiluminescence for calcitonin testing and electroluminescence for thyroitropin receptor antibodies (TRAbs (Advia Centaur XPT, Siemens, Germany and Atellica IM 1600, Siemens, Germany)). Biochemical testing was performed on a Vitros 7600 analyzer (MYCO Instrumentation Inc, Bonney Lake, WA, USA). Genetic testing, although recommended, was not performed because all patients denied it due to financial reasons. All patients signed an informed consent form that was approved by the Timișoara County Hospital ethics committee. Moreover, this study was carried out in accordance with the Declaration of Helsinki’s Ethical Principles for Medical Research, agreeing to the use of their data that were collected during admission.

## 3. Case Series Presentation

### 3.1. Case 1

#### 3.1.1. Case Presentation

A 63-year-old female patient was admitted to the Endocrinology Department of the County Hospital Timișoara, Romania, in February 2015 with dysphagia, anterior neck discomfort, and palpitations. She was diagnosed in 2009 with a multinodular goiter with an euthyroid status. She denied any family history of cancer or Multiple Endocrine Neoplasia (MEN) syndrome. Her recent medical history revealed that she was diagnosed with paroxysmal atrial fibrillation and osteopenia shortly before the current admission. She was on antiarrhythmic treatment with Propafenone and oral anticoagulant therapy with warfarin.

Upon admission, the patient’s general physical examination revealed a BMI of 34.81 kg/m^2^, regular heart rate of 82 bpm, and blood pressure of 110/60 mmHg. A thyroid examination indicated a diffusely enlarged thyroid gland without any palpable nodules.

#### 3.1.2. Thyroid Ultrasound

**Figure 1:**
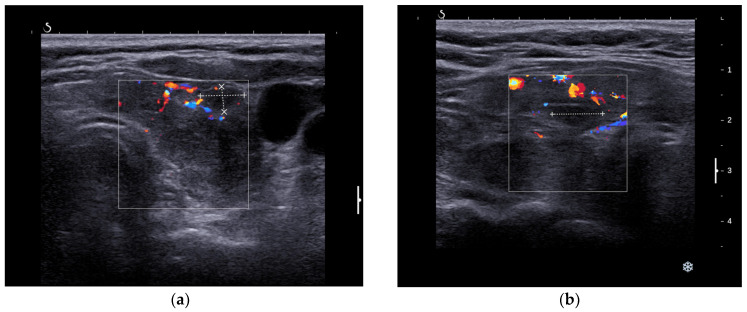
(**a**) LTL with hypoechoic, inhomogeneous parenchyma, intense vascularity with a solid, hypoechoic nodule with a diameter of 8/5/9 mm, and with perinodular vascularity and irregular borders. (**b**) RTL with a solid, hypoechoic nodule with a diameter of 8/6/10 mm, without halo, and with perinodular vascularity.

**Figure 2:**
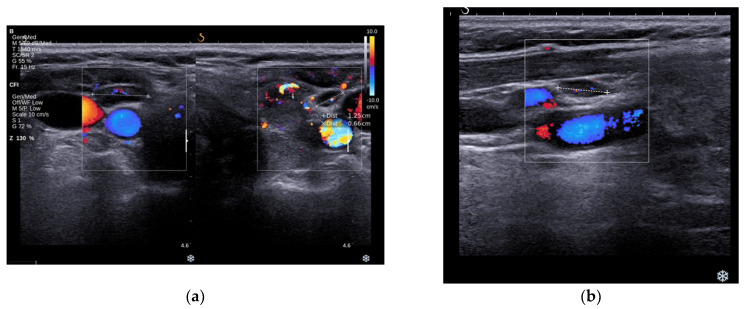
(**a**) Right inflammatory adenopathy with ⌀ of 12 mm. (**b**) Left inflammatory adenopathy with ⌀ of 7.5 mm.

A high-resolution ultrasound examination of the anterior neck revealed a diffuse enlargement (40.6 mL) of the thyroid with hypoechoic, inhomogeneous parenchyma and intense vascularity, as well as two solid hypoechoic nodules with perinodular vascularity and irregular borders in both thyroid lobes (ACR-TIRADS 5). The largest nodule was in the left thyroid lobe (LTL) with a diameter of 8/5/9 mm (Figure 1a). The second one was positioned in the right thyroid lobe (RTL) with a diameter of 8/6/10 mm (Figure 1b). The examination also revealed bilateral adenopathy without any suspicious ultrasonographic features for a malignancy (Figure 2a,b).

#### 3.1.3. Pre-Operative Laboratory Tests and Treatment

During the current admission, paraclinical evaluations confirmed overt thyrotoxicosis with thyroid-stimulating hormone (TSH) = 0.002 mUI/L (0.55–4.78 mUI/L), FT4 = 86.49 pmol/L (11.50–22.7 pmol/L), and FT3 = 16.56 pmol/L (3.54–6.47 pmol/L). TRAb levels were increased at 11.7 U/L (<1.75 U/L) (Table 1). Prompt treatment with Thiamazole 30 mg per day was started, with decreasing dosages over the next few weeks. Due to the suspicious appearance of the nodules on an ultrasound, plasma calcitonin levels were tested and revealed an increased value of 49.10 pg/mL (<5 pg/mL). Carcinoembryonic antigen (CEA) was within normal parameters at 1.93 ng/mL (<5 ng/mL). After 6 months of treatment with Thiamazole, laboratory tests revealed an euthyroid status (Table 1).

Suspecting MTC and due to the patient severe dysphagia complaints, a total thyroidectomy was performed in May 2015 after achieving an euthyroid status. Prior to surgery, the patient tested negative for hyperparathyroidism and pheochromocytoma, with normal values for PTH, total calcium levels, and serum methanephrines and normetanephrines. Chest and abdominal CT scans revealed no signs of metastatic disease. Post-surgery, treatment with levothyroxine 100 mcg/day was initiated.

#### 3.1.4. Histological Examination

The pathology report revealed LTL with characteristic GD histologic alterations and a small nodular lesion of 0.9/0.7 cm with sparse cellularity and amyloid deposition, microcalcifications, and atypical cellularity. Immunohistochemistry indicated positive expression for calcitonin. No tumor foci were found in the RTL. The final diagnosis was MTC with amyloid stroma, pT1 Nx R0.

#### 3.1.5. Post-Operative Evaluation and Management

In August 2016, one year after surgery, investigations showed normal TSH and FT4 levels on supplementation with levothyroxine 100 mcg/day. An anterior neck ultrasound revealed no signs of recurrence or persistent disease. Abdominal and chest CT were performed, revealing no signs of metastatic disease or tumor recurrence. Calcitonin levels were nondetectable (<5 pg/mL). RET testing was recommended but the patient refused due to financial reasons. The patient made a full recovery without further complications.

### 3.2. Case 2

#### 3.2.1. Case Presentation

A 79-year-old female was admitted to our department in March 2022 for evaluation due to fatigue. She was diagnosed with GD in 2011, presenting significant weight loss and frequent palpitations at the time. Initial laboratory investigations indicated clinical hyperthyroidism with positive TRAb assay, and she was started on Methimazole, which she continued at the time of the present admission. In 2021, an ultrasound of the anterior neck region revealed multiple thyroid nodules with suspicious features. Serum calcitonin was extremely elevated at 698 pg/mL (<5 pg/mL). Her family history was unremarkable.

The patient was also diagnosed with extrasystolic ventricular arrhythmia and osteopenia, receiving antiarrhythmic treatment with Sotalol and anti-osteoporotic treatment with bisphosphonates and vitamin D supplementation.

The clinical exam revealed a BMI of 28.1 kg/m^2^, a regular heart rate of 76 bpm, and blood pressure of 125/85 mmHg. Thyroid examination did not reveal the presence of goiter.

#### 3.2.2. Thyroid Ultrasound

**Figure 3:**
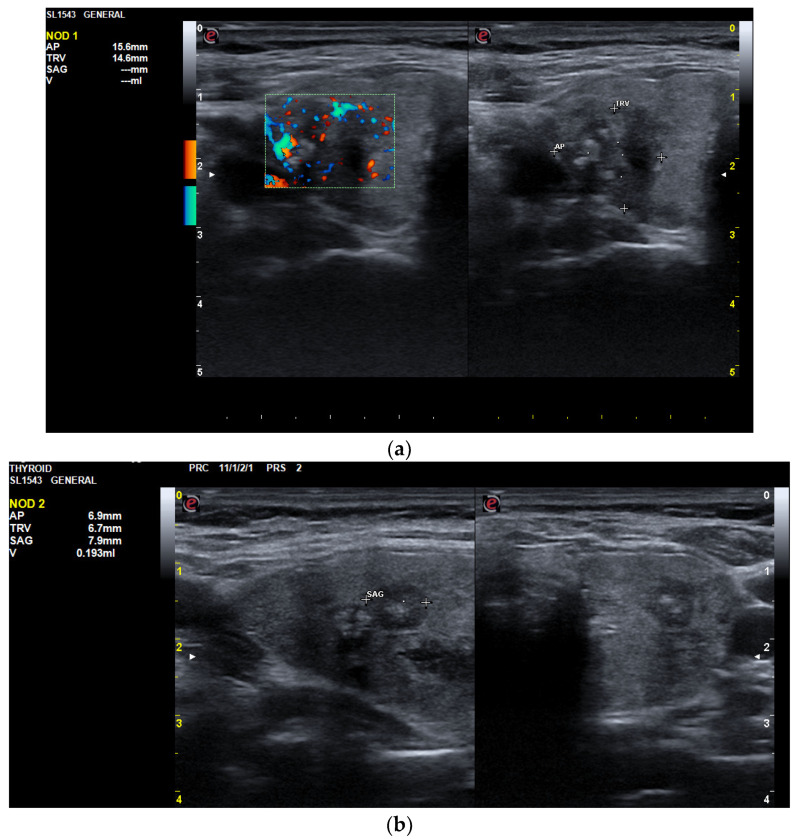
(**a**) RTL: with a solid, hypoechoic nodule with a diameter of 14.6/15.6/22 mm, internal gross calcifications, and ill-defined margins. (**b**) LTL with two solid nodules with a diameter of 7/7/8 mm and 5.7/6.9/6 mm with internal gross calcifications and ill-defined margins.

An ultrasound examination revealed an increased thyroid volume of 26.4 mL with hypoechoic and inhomogeneous parenchyma, along with three solid hypoechoic nodules with internal gross calcifications and ill-defined margins (ACR-TIRADS 5). The largest nodule was in the RTL with a diameter of 14.6/15.6/22 mm. (Figure 3a) The other two nodules were smaller than 1 cm and positioned in the LTL with similar features to the dominant nodule (Figure 3b).

#### 3.2.3. Pre-Operative Investigations

Before treatment with Thiamazole, laboratory tests revealed overt thyrotoxicosis. After six months of treatment, laboratory tests indicated increased TSH of 5.76 mUI/L (0.55–4.78 mUI/L) with normal FT4 13.4 pmol/L and FT3 4.55 pmol/L. Anti-TPO and TG antibodies were elevated at >1300 UI/mL and 101 UI/mL, respectively. TRAb values were also elevated at 4.49 U/L (<1.75 U/L). Calcitonin was markedly increased at 832 pg/mL (<5 pg/mL) and CEA was 12.69 ng/mL (<5 ng/mL) (Table 2). Chest and abdominal CT revealed no tumoral involvement.

A CT examination of the neck and chest revealed diffuse thyroid enlargement with bilateral calcifications and no tumoral involvement in the pleuro-pulmonary region. Given these findings and the high calcitonin value, MTC was suspected, and a total thyroidectomy with bilateral lymphadenectomy of the 3rd and 4th compartments was performed. Prior to surgery, the patient tested negative for hyperparathyroidism and pheochromocytoma, with normal values for PTH, total calcium levels, and serum methanephrines and normetanephrines. After the surgery, levothyroxine supplementation therapy was initiated with 100 mcg/day.

#### 3.2.4. Histological Examination

The pathology report revealed a solid yellow tumor of 2.2/1.08 cm at the upper pole of the RTL and a left lobectomy specimen with inhomogeneous parenchyma and numerous yellow cysts. Microscopy indicated an insular and solid growth pattern with amyloid deposition in the tumor stroma. The conclusion was MTC pT2 (m) N0 with bilateral tumor involvement. The parenchyma suggested alterations indicative of GD.

#### 3.2.5. Post-Operative Evaluation and Management

Two months post-surgery, the patient had normal TSH and FT4 levels on levothyroxine supplementation. Calcitonin levels decreased significantly to 113 pg/mL (<5 pg/mL). An anterior neck ultrasound showed no signs of recurrence or persistence. The patient made a full recovery without signs of hypoparathyroidism or recurrent laryngeal nerve damage. Abdominal, neck, and chest CT scans were performed, revealing no signs of metastatic disease or tumor recurrence. RET testing was recommended but the patient refused due to financial reasons.

### 3.3. Case 3

#### 3.3.1. Case Presentation

A 39-year-old female was admitted to our department in May 2014, complaining of dysphagia, intermittent dyspnea, and fatigue. She was diagnosed with GD during her first pregnancy and successfully treated with antithyroid medication for 5 years. After the treatment was stopped, she developed a recurrence of the disease and was again started on Thiamazole, which was continued throughout her second pregnancy and during the current admission. Her family history was unremarkable.

The clinical exam revealed a BMI of 25.1 kg/m^2^, a regular heart rate of 71 bpm, and blood pressure of 140/85 mmHg. A thyroid examination revealed a diffusely enlarged goiter.

#### 3.3.2. Thyroid Ultrasound

**Figure 4:**
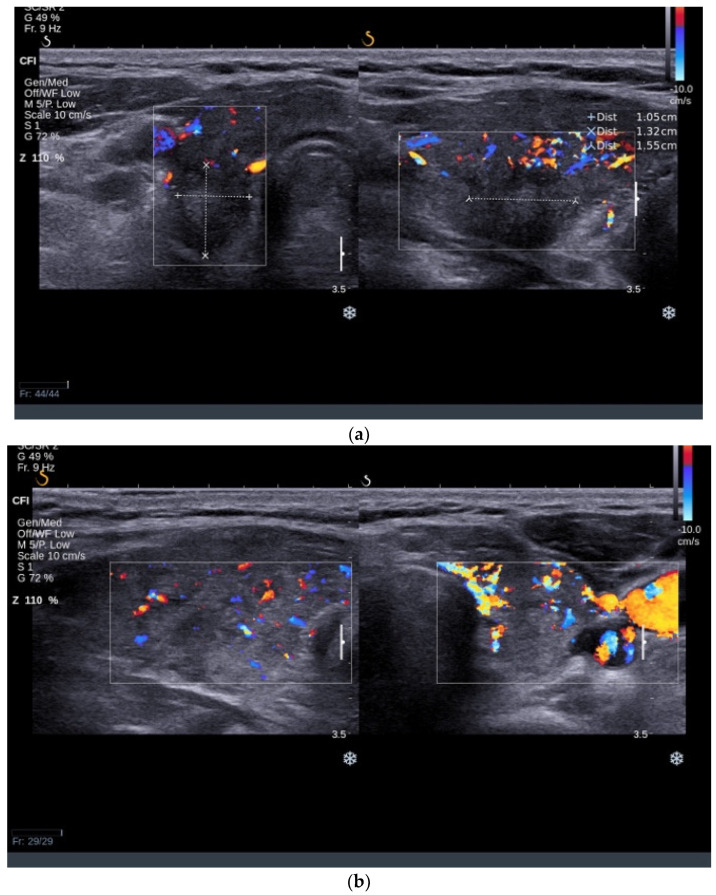
(**a**) RTL with a solid, hypoechoic nodule of 10/13/15 mm diameter. (**b**) Hypoechoic and inhomogeneous parenchyma of the LTL with multiple small nodular lesions.

**Figure 5:**
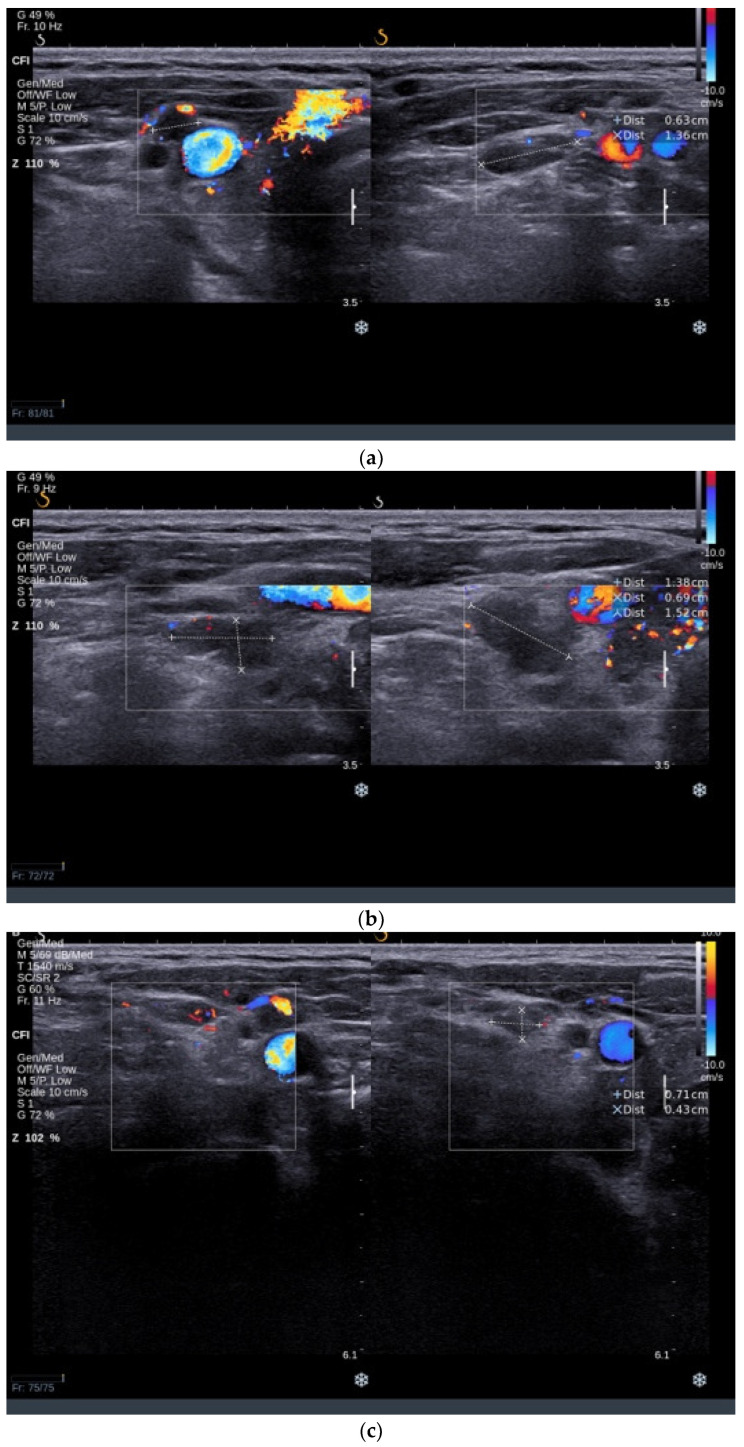

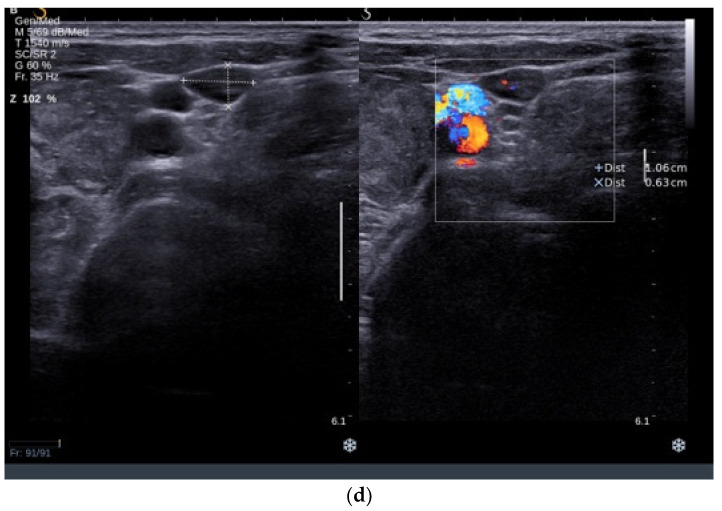
Parts (**a**–**d**) showing multiple enlarged lymph nodes with round shape and absent central hilar vascularity.

An ultrasound examination revealed an increased thyroid volume of 38 mL with hypoechoic and inhomogeneous parenchyma with increased vascularity. A solid hypoechoic nodule with a diameter of 10/13/15 mm was found in the RTL with ill-defined margins and no vascularity, ACR-TIRADS 5 (Figure 4a). Multiple small solid nodular lesions with similar features were found in the LTL (Figure 4b). Bilateral latero-cervical lymph nodes were enlarged with a round shape, absent central hilum, and subcapsular vascularity (Figure 5a–d), raising concerns about the nature of the nodular lesions present in the thyroid.

#### 3.3.3. Pre-Operative Investigations

Laboratory tests indicated an euthyroid status with TSH of 1.7 mUI/L (0.55–4.78 mUI/L), normal FT4 15.7 pmol/L, and FT3 4.3 pmol/L. Anti-Thyroperoxidase antibodies were elevated at >1300 UI/mL, while anti-TG antibodies were within normal ranges. TRAb values were not determined at the time of the evaluation but were increased in a previous evaluation (previously 13.7 U/L). Given the suspicious features of the adenopathies, serum calcitonin was evaluated and found to be markedly increased at 2032 pg/mL (<5 pg/mL) (Table 3). Neck, chest, and abdominal CT scans revealed no tumoral involvement.

Suspecting MTC, a total thyroidectomy with bilateral lymphadenectomy of the 3rd and 4th compartments was performed. The patient tested negative for hyperparathyroidism and pheochromocytoma, with normal values for PTH, total calcium levels, and serum methanephrines and normetanephrines. RET testing was recommended but the patient refused due to financial reasons.

After the surgery, levothyroxine supplementation therapy was initiated with 100 mcg/day.

#### 3.3.4. Histological Examination

The pathology report revealed an enlarged RTL with a solid yellow tumor of 1.5/1/1 cm and a left lobectomy specimen with multiple small nodular lesions below 1 cm. Microscopy indicated an insular and solid growth pattern with amyloid deposition in the tumor stroma. The conclusion was MTC pT2 (m) N1 with bilateral tumor involvement. The parenchyma suggested alterations indicative of GD.

#### 3.3.5. Post-Operative Evaluation and Management

Six months post-surgery, the patient had a normal euthyroid status on levothyroxine supplementation of 125 mcg/day. The patient developed permanent hypoparathyroidism after the surgery. Calcitonin levels decreased but remained elevated at 150 pg/mL (<5 pg/mL). Abdominal, neck, and chest CT scans were performed, revealing no signs of metastatic disease or tumor recurrence. An anterior neck ultrasound revealed no signs of recurrence or persistent disease.

The patient was lost to follow-up and did not return for further check-ups.

## 4. Discussion

In 1937, Means et al. proposed that hyperthyroidism might serve as a protective mechanism against the development of thyroid cancer [11]. However, later studies, including one by Shapiro et al., demonstrated an increased incidence of thyroid cancer, showing an 8.7% occurrence in GD patients [12]. The true incidence remains controversial, with reports ranging from 0.5% to 15.0% and an annual incidence of 17.5 per 100.000, which is significantly higher than the 0.5 to 8.0 per 100.000 incidence reported for the general euthyroid population [13,14,15].

Recent research indicates that thyroid cancer in GD patients has a worse prognosis. In a study with 22 patients, tumors in GD patients were often larger (3.3 ± 1.8 cm vs. 1.0 ± 0.7 cm), multifocal (46.1% vs. 0%), locally invasive (61.5% vs. 11.1%), or metastatic (23.0% vs. 0%) [16]. This could be attributed to the presence of TRAbs, which promote thyroid cell growth and angiogenesis by activating the IGF system and vascular endothelial growth factor (VEGF) [17].

Studies have shown that the most common histological type of thyroid cancer in GD patients is papillary carcinoma (80%), followed by follicular thyroid carcinoma (10%) [6,18]. Medullary thyroid carcinoma (MTC) is diagnosed in 0.6% of GD patients [19]. MTC is an aggressive form of thyroid cancer that originates from the parafollicular C cells of the thyroid gland. About 75–80% of MTC cases occur sporadically, while the rest are familial and are associated with MEN 2A and MEN 2B and familial medullary thyroid cancer syndrome [20]. MTC predominantly affects females (59.8%) and younger adults under 65 years (69.2%) [21]. Surgical resection remains the primary treatment as MTC does not respond to radioactive iodine therapy or chemotherapy [22]. Metastases to the bone, liver, lung, or brain are present in 10% of cases at diagnosis, making early detection crucial [20].

A systematic review reported only 15 cases of MTC diagnosed in GD patients, mostly between 1995 and 2005. Most patients were asymptomatic at diagnosis, with a higher prevalence in females. Only a third of the tumors were larger than 2 cm [9]. Our case series aligns with these findings, with all patients being female, with no symptoms of malignancy prior to diagnosis, and presenting with thyroid nodules smaller than 2 cm on ultrasounds.

Elevated plasma calcitonin is a critical marker for MTC presence and extent. The European Thyroid Association recommends routine calcitonin testing for all thyroid nodules with suspicious ultrasound features, while the American Thyroid Association suggests that pre-operative diagnosis should rely on FNAB. Studies have shown that serum calcitonin testing has a higher sensitivity for MTC diagnosis than cytological examination [22].

Serum calcitonin testing is also essential for validating treatment success. Both the American Thyroid Association (ATA) and the National Comprehensive Cancer Network (NCCN) guidelines recommend calcitonin and CEA testing 2–3 months post-treatment to confirm curative surgery. Some authors suggest testing sooner as calcitonin values may become undetectable within one month after surgery [23]. Our findings in the second case support this as calcitonin levels significantly decreased one month after surgery but were not below the reference threshold, even after excluding recurrent or metastatic disease, supporting the current guidelines for a post-operative evaluation 2–3 months after the procedure.

The association between GD and MTC is likely incidental. Some studies have suggested that TSH receptors might be present in parafollicular C cells, allowing TRAbs to influence the growth of C cells with genetic and epigenetic mutations. Somatic mutations in the RET gene, identified in 40–50% of sporadic MTC cases, are linked to a worse prognosis. Additionally, increased levels of interleukins and TNF-alpha in GD might stimulate endothelin-1, contributing to carcinogenesis and angiogenesis, as its overexpression has been demonstrated in MTC patients [24].

Total thyroidectomy with lymph node dissection is the preferred treatment for these patients with hyperthyroidism [25] that are at a higher risk of post-operative complications, including transient hypoparathyroidism (28.1% vs. 13.2%; *p* < 0.01) and local recurrence (5.7% vs. 2.5%) [25,26,27].

## 5. Conclusions

The longstanding hypothesis that hyperthyroidism may confer a protective advantage against thyroid cancer was thoroughly discredited.

This paradigm shift mandates a heightened vigilance in the monitoring and management of thyroid nodules in patients with hyperthyroidism, especially those diagnosed with GD, to ensure the prompt detection and treatment of malignancies.

We strongly recommend incorporating serial calcitonin testing into the routine follow-ups of GD patients with a nodular goiter, particularly when suspicious features are identified on an ultrasound. Timely diagnosis and treatment are vital for the survival of patients with MTC. Given the increased aggressiveness and complication rates of cancers in GD, we consider that a high level of suspicion and detailed evaluations are essential for GD patients presenting with thyroid nodules.

## Figures and Tables

**Table 1:** Pre-operative laboratory investigations.

Test	Value	Reference Range
**At admission**		
TSH	0.002 mUI/L	0.55–4.78 mUI/L
FT3	16.56 pmol/L	3.54–6.47 pmol/L
FT4	86.49 pmol/L	11.50–22.7 pmol/L
TRAB	11.7 U/L	<1.75 U/L
ATPO	>1300 U/L	0–60 U/L
Calcitonin	49.10 pg/mL	<5 pg/mL
CEA	1.93 ng/mL	<5 pg/mL
**After treatment with Thiamazole**		
TSH	0.9 mUI/L	0.55–4.78 mUI/L
FT3	4.7 pmol/L	3.54–6.47 pmol/L
FT4	15.6 pmol/L	11.50–22.7 pmol/L

TSH—thyroid-stimulating hormone, FT3—free T3, FT4—free T4, TRAB—thyroid-stimulating antibodies, ATPOs—antithyroid peroxidase antibodies, CEA—carcinoembryonic antigen.

**Table 2:** Pre-operative laboratory investigations after treatment with Thiamazole.

Test	Value	Reference Range
TSH	5.76 mUI/L	0.55–4.78 mUI/L
FT3	4.55 pmol/L	3.54–6.47 pmol/L
FT4	13.4 pmol/L	11.50–22.7 pmol/L
TRAB	4.49 U/L	<1.75 U/L
AFP	0 ng/mL	0.0–1.8 ng/mL
Calcitonin	832 pg/mL	<5 pg/mL
CEA	12.69 ng/mL	<5 ng/mL

TSH—thyroid-stimulating hormone, FT3—free T3, FT4—free T4, TRAB—thyroid-stimulating antibodies, CEA—carcinoembryonic antigen, AFP—alpha-fetoprotein.

**Table 3:** Pre-operative laboratory investigations.

Test	Value	Reference Range
TSH	1.7 mUI/L	0.55–4.78 mUI/L
FT3	4.3 pmol/L	3.54–6.47 pmol/L
FT4	15.7 pmol/L	11.50–22.7 pmol/L
ATPO	>1300 U/L	0–60 U/L
ATG	3 ng/mL	0–45 U/mL
Calcitonin	2032 pg/mL	<5 pg/mL

TSH—thyroid-stimulating hormone, FT3—free T3, FT4—free T4, TRAB, ATPO—antithyroid peroxidase antibodies, ATG—antithyroglobulin antibodies.

## Data Availability

The patients’ data are unavailable due to privacy or ethical restrictions.
